# Intratumoral interleukin-6 predicts ascites formation in patients with epithelial ovarian cancer: A potential tool for close monitoring

**DOI:** 10.1186/s13048-015-0183-x

**Published:** 2015-08-19

**Authors:** Samar Masoumi-Moghaddam, Afshin Amini, Ai-Qun Wei, Gregory Robertson, David L. Morris

**Affiliations:** Department of Surgery, St George Hospital, The University of New South Wales, Gray Street, Kogarah, Sydney, NSW 2217 Australia; Department of Orthopedic Surgery, St. George Hospital, The University of New South Wales, Gray Street, Kogarah, Sydney, NSW 2217 Australia; Department of Gynecology Oncology, St George Hospital, The University of New South Wales, Gray Street, Kogarah, Sydney, NSW 2217 Australia

**Keywords:** Ascites, Chemorefractoriness, Disease free survival, Epithelial ovarian cancer, Interleukin-6, Overall survival

## Abstract

**Background:**

The implication of IL-6 in the pathogenesis of epithelial ovarian cancer (EOC) is well documented. Accordingly, the clinicopathological significance of this cytokine in patients’ ascites fluid or serum has largely been investigated. Since the main source of IL-6 secreted into the biological fluids is the tumor tissue, this study was designed to investigate the status and possible clinical relevance of the IL-6 expression in an array of EOC tissue specimens.

**Methods:**

Tissue samples obtained from ninety-eight consecutive patients with EOC were studied using immunohistochemistry. Clinicopathological characteristics and treatment related factors were collected from patient files. The relationship between the expression of the protein of interest and the study endpoints of disease-free survival (DFS) and overall survival (OS) were analyzed using the Kaplan-Meier method. For evaluating the predictive value of IL-6, logistic regression and cox proportional hazards models were employed.

**Results:**

An upregulation of IL-6 expression was observed in EOC tissues as compared with the normal samples (*p* < 0.0001). As regards the clinical relevance, IL-6 failed to predict OS, DFS and response to the platinum-based chemotherapy in EOC patients. In multivariate analysis, however, IL-6 was identified as an independent predictive factor for the development of post-treatment ascites (p:0.033).

**Conclusions:**

Having the capability to predict the ascites formation, IL-6 might serve as a biomarker and a useful tool in EOC for monitoring purposes. IL-6 targeting for the prevention of the ascites development is a potential avenue for further investigation.

## Background

Interleukin-6 (IL-6) is a classic pro-inflammatory cytokine associated with a variety of pathological conditions, including cancer. Evidence supports the role of IL-6 in the pathophysiology of epithelial ovarian cancer (EOC) where the presence of an immunosuppressive network protecting tumor from immune system is also involved in EOC growth and progression [1–3]. While contributing to the follicle development in normal ovaries and the regulation of chronic inflammation, IL-6 can be involved in the provision of a cellular microenvironment beneficial to cancer cell growth, proliferation, migration and survival. This can result from direct effects on tumor cell biology along with the regulation of the immune system components [4, 5]. *In vitro* studies have confirmed the production of IL-6 by normal and neoplastic ovarian epithelium [6, 7]. The association of IL-6 with EOC carcinogenesis and progression [8], as well as with the enhancement of EOC cell survival, resistance to chemotherapy [9] and invasiveness [10] has also been reported.

In the present study, we investigated the expression status of the intratumoral IL-6 in EOC tissue samples compared with normal ovarian tissue and evaluated a possible clinical relevance with regard to survival, response to the platinum-based chemotherapy and ascites formation. As a result, we observed a significant upregulation of IL-6 in the EOC tissue. Moreover, we identified IL-6 expression status of EOC tumor as an independent predictive biomarker for ascites formation in patients receiving adjuvant carbotaxol treatment.

## Patients and methods

### Clinical cases and surgical samples

Initially, institutional review board approval for this analysis was obtained from South Eastern Sydney and Illawarra Area Health Service Human Research Ethics Committee-Central Network (EC00135). Upon giving informed written consents, ninety-eight consecutive patients with primary epithelial ovarian cancer who had undergone standard surgical procedure (staging laparotomy/cytoreductive surgery) and adjuvant systemic chemotherapy (paclitaxel + carboplatin) between January 2001 and December 2012 at two specialized centers (St. George Hospital; St George Private Hospital, Sydney, Australia) were included in the study. A confirmatory review of pathology was performed. Ovarian neoplasms were histologically classified according to the World Health Organization (WHO) classification system [11]. The final staging of the disease was determined on the basis of a combination of surgical and pathological findings in accord with the Federation of Gynecology and Obstetrics (FIGO) guidelines [12]. Clinicopathological characteristics of the participants are shown in Table 1.

**Table 1:** Clinicopathological characteristics of the participants

Characteristic		Categorization	Patients (*n* = 98)
Age (years)	Range: 35–84	≤50	16
	Median: 62	>50	82
Histological subtype		High-grade serous	63
		Low-grade serous	17
		High-grade endometrioid	1
		Low-grade endometrioid	2
		Mucinous	2
		Clear cell	5
		Others	8
FIGO stage		I-II	14
		III-IV	84
Extent of residual tumour		None	46
		<1 cm	35
		1–2 cm	0
		>2 cm	17
Clinical response to platinum chemotherapy		Sensitive	77
		Resistant	21
post-treatment ascites		Yes	40
		No	58

### Immunohistochemical staining

For immunohistochemical study, five-micrometer sections were prepared from the paraffin blocks of the patients and immunostained as described previously [13]. Briefly, the sections were deparaffinized and incubated with 3 % hydrogen peroxide and DAKO blocking buffer, subsequently. This was followed by overnight incubation at 4 °C with mouse monoclonal primary antibody (Santa Cruz) and then with appropriate secondary antibody using EnVision Plus kit (DAKO). The sections were then counterstained with hematoxylin. Tonsil tissue was used as the positive control. As regards the negative control, the same tissue as our positive control was used but the primary antibodies were replaced with the primary antibody diluents.

### Immunohistochemical scoring

To evaluate the staining of the epithelial cells, a semi-quantitative scoring method was performed according to Mattern et al. and Terris et al. [14, 15]. This scoring method enables the determination of both the intensity of the immunosignal and the percentage of cells showing positive staining. The percentage of positive cells was scored as: no positive cells (0); 1–25 % (1); 26–50 % (2); and 50 % > (3). The intensity of the staining was scored as: no staining (0); weak (1); moderate (2); strong (3). Following the evaluation by two observers blinded to patient outcome, the immunohistochemical score was calculated as follows, yielding a range of values between 0 and 9: immunohistochemical score = [percentage of positive cells] × [intensity of staining].

### Statistical analysis

All statistical analyses were conducted using the statistical package SPSS, version 22 (SPSS Inc., USA). Student *t*-test was used for comparing the actual difference between two means. Spearman correlation coefficient testing was performed to evaluate the associations between the clinicopathological parameters and the expression of IL-6. The binary cut-off point was identified using the Classification and Regression Tree (CART) algorithm. According to the cut-off point determined, the immunohistochemical scores were classified as either low (score ≤ 3.5) or high (score > 3.5). The relationship between the IL-6 expression and the study endpoints of disease-free survival (DFS) and overall survival (OS) were individually analyzed using the Kaplan-Meier method. Univariate and multivariate logistic regression analyses were conducted to ascertain the effect of the studied cytokine and other clinicopathological variables on the likelihood of the development of post-treatment ascites or chemotherapy refractory disease. A p value of < 0.05 was considered statistically significant for all analyses.

## Results

### EOC tumors express higher levels of IL-6

**Fig. 1:**
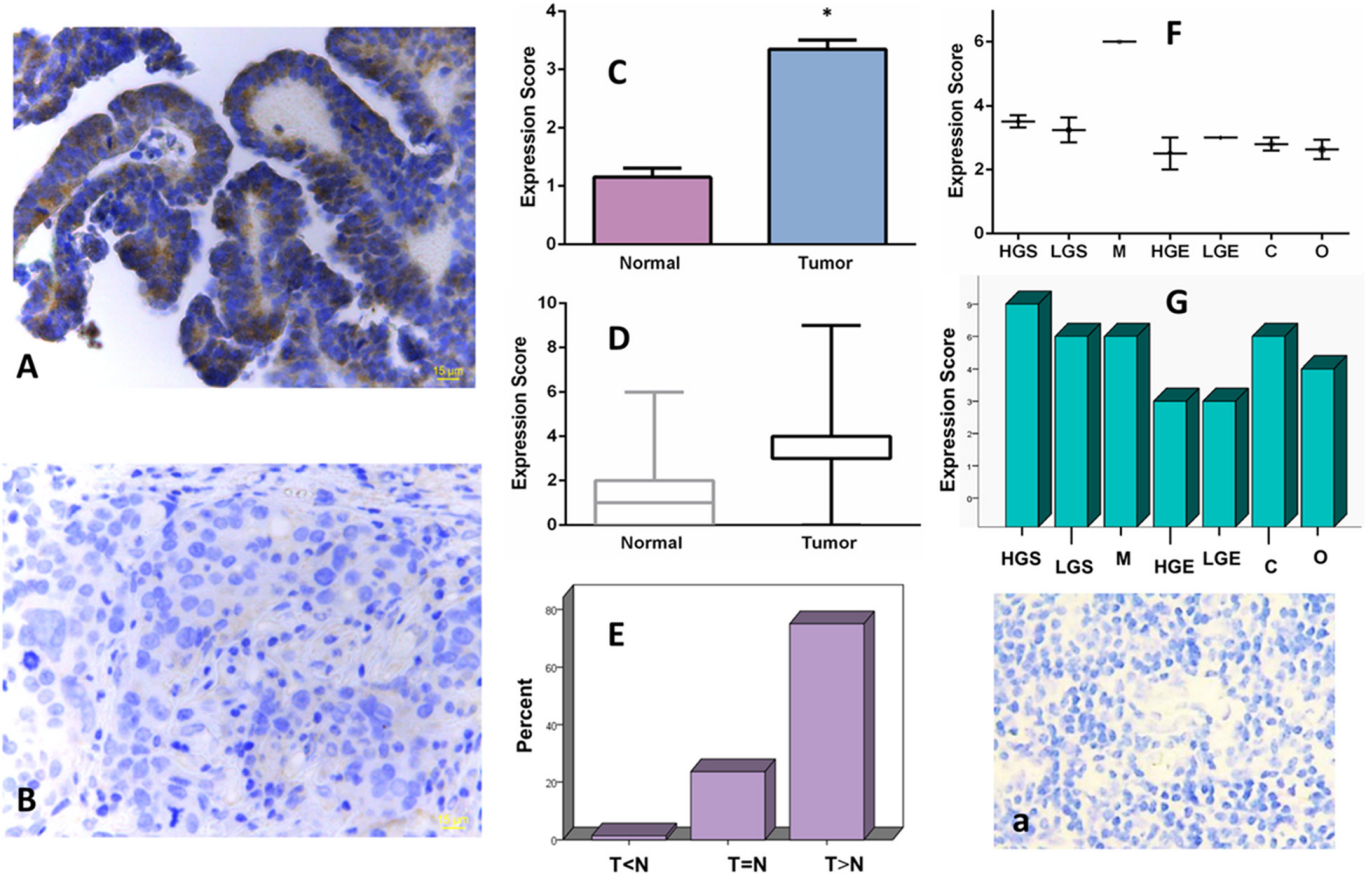
Immunohistochemical analysis of IL-6 expression in human epithelial ovarian cancer (EOC) tissue Representative micrographs show high (**A**) and low (**B**) levels of the immunohistochemical expression of IL-6 in the high-grade serous EOC tissue (magnification = 40x) (**a**) represents the negative control. Graphs (**C**) and (**D**) demonstrate the upregulation of IL-6 in EOC as compared with the normal ovarian tissue. Data are presented as mean expression score ± SE (**C**) and maximum and minimum expression score (**D**). Significant values (<0.05) are marked by asterisks. Graph (**E**) indicates the percentage of cases with higher (T > N), lower (T < N) or equal (T = N) intratumoral levels of IL-6 when the protein expressions in tumoral and matched normal ovarian tissues were compared. Graph (**F**) shows the mean expression scores of IL-6 in different subgroups of EOC as mean expression score ± SE. Graph (**G**) depicts maximum and minimum expression scores of IL-6 in different subgroups of EOC. HGS: high grade serous; LGS: low grade serous; M: mucinous; HGE: high grade endometrioid; LGE: low grade endometrioid; C: clear cell; O: others. Magnification = 40x

Comparing the expression status of IL-6 in EOC tissues with that in normal samples, we found significantly higher levels of the IL-6 expression in tumor tissues (*p* < 0.0001). Mean expression scores were 3.35 ± 0.15 and 1.15 ± 0.15 for tumor and normal biopsies, respectively. When the IL-6 immunohistochemical scores of matched normal and cancer tissues were compared, we observed higher levels of the IL-6 expression in seventy-five percent of tumor tissues (Fig. 1).

### IL-6 predicts development of post-treatment ascites

In our cohort, while 35 cases had no history of ascites either at the time of diagnosis or after treatment, ascites was present in 63 patients. Mean IL-6 expression scores in the ascites group (3.59 ± 0.19) were significantly higher than those in the non-ascites group (2.91 ± 0.24, p:0.039). Subgroup analysis did not indicate a statistically significant difference in mean IL-6 expression scores between patients who had ascites at the time of diagnosis (3.56 ± 0.22) and those without ascites (3.11 ± 0.21, p:0.154). On the other hand, the comparison of mean IL-6 expression scores between patients who did and did not develop ascites after treatment (3.85 ± 0.26 and 3.00 ± 0.18, respectively) revealed significantly higher levels of IL-6 expression in the ascites positive group (p:0.007). When the association between the expression of IL-6 and the history of ascites was evaluated, a direct significant correlation was found between the IL-6 expression and post-treatment ascites formation (p:0.034, correlation coefficient = 0.214).

The predictive value of IL-6 expression with regard to the development of post-treatment ascites was further evaluated employing logistic regression analysis. Univariate test revealed the significance of the IL-6 expression for predicting the development of post-treatment ascites (*HR* = 0.39; 95 % CI, 0.16-0.94; p 0.036). Stage (*HR* = 0.08; 95 % CI, 0.01–0.67; p:0.020), ascites at diagnosis (*HR* = 0.23; 95 % CI, 0.09–0.55; p:0.001) and refractory disease (*HR* = 0.15; 95 % CI, 0.05–0.46; p:0.001) also appeared to be of predicting value among other clinicopathological features. In multivariate logistic regression analysis, IL-6 (*HR* = 0.31; 95 % CI, 0.10–0.91; p:0.033), ascites at diagnosis (*HR* = 0.22; 95 % CI, 0.07–0.66; p:0.007) and refractory disease (*HR* = 0.09; 95 % CI, 0.02–0.36; p:0.001) retained their values as independent predictors of post-treatment ascites in EOC patients whereas stage (p:0 .075) failed to show an independent predictive value.

### IL-6 has no predictive value for overall survival and disease free survival

In the statistical analysis of overall survival (OS), the difference in median OS of IL-6 high- and low-expressing groups (3.8 and 3.9 years, respectively) as well as in mean OS of these two groups (4.7 and 5.3 years, respectively) was not statistically significant (p:0.646). With respect to disease free survival (DFS) of IL-6 high- and low-expressing groups, there was no statistically meaningful difference (p:0.30) between the median (22 vs 30 months, respectively) or mean values (41.5 vs 50 months, respectively) of the two groups, either. Cox analysis of the results consistently failed to show a predictive value for IL-6 with regard to survival (OS, p:0.646; DFS, p:0.301).

### IL-6 does not predict platinum-based refractory disease

Finally, we investigated whether the expression status of IL-6 in surgical specimens has the potential to predict response to the platinum-based chemotherapy. When patients who later showed resistance to carbotaxol chemotherapy were accordingly compared with those who remained sensitive to the same regimens, mean IL-6 expression scores of the refractory (3.19) and sensitive (3.39) groups turned out to be similar (p:0.605). Our logistic regression analysis consistently demonstrated that IL-6 expression fails to predict response to the platinum-based treatment (p:0.760).

## Discussion

IL-6 is known as a pleotropic cytokine implicated in EOC carcinogenesis. It influences EOC growth and development through direct and indirect effects on tumor cells or their microenvironment, respectively [4, 5]. As such, IL-6 has been indicated to promote EOC cell proliferation, migration, invasion, survival and resistance to chemotherapeutic agents [9, 10, 16]. Reports show that IL-6 contributes to the development of EOC-induced malignant ascites [8]. Here, we demonstrated that IL-6 was expressed at higher levels in human EOC tissues than in normal ovarian tissues. In agreement, increased tumoral expression [17] and high serum [18, 19] or ascetic [20] levels of IL-6 have been reported in patients with EOC. Accordingly, clinicopathological relevance of intratumoral, serum or ascitic IL-6 in EOC has been investigated [18–25]. Plewka et al. [26] observed that malignant serous tumors had higher expression levels of IL-6 as compared with serous borderline and benign lesions. Guo et al. [27] reported a significant difference in immunohistochemical expressions of IL-6 among the metastatic, drug-resistant recurrent tumors, and matched primary tumors, with more staining density and positivity observed in the drug-resistant and metastatic tumors. In another study by Coward et al. [28], intensity of IL-6 staining in malignant cells was found to be significantly associated with poor prognosis. Similarly, a link between high levels of serum or ascitic IL-6 and unfavorable clinical outcome has been reported. In this regard, serum IL-6 has shown significant association with tumor burden, clinical disease status, and survival [21, 22], as well as prognostic value [25], in EOC. In a study by Scambia et al. [29], higher levels of serum IL-6 was found in patients unresponsive to chemotherapy. In line with these findings, Cohen et al. [30] found that cisplatin treatment of EOC cells upregulated IL-6, and IL-6 inhibition, on the other hand, resulted in significant sensitization to cisplatin, suggesting that IL-6 could contribute to the induction of platinum resistance in EOC. In agreement, Wang et al. found that both exogenous and endogenous IL-6 could induce cisplatin and paclitaxel resistance in vitro via increased expression of both multidrug resistance-related genes and apoptosis inhibitory proteins, as well as activation of extracellular signal-regulated kinases (ERK) and Akt signaling [9]. They also demonstrated that IL-6 might contribute to the refractoriness of EOC to tamoxifen [31]. In our study, however, intratumoral IL-6 showed neither dependent nor independent predicting value for OS, DFS or response to the platinum-based chemotherapy. In line with our results, Plante el al [20] indicated that serum and ascites IL-6 levels did not correlate statistically with OS. In contrast to our observation, where almost equal intratumoral IL-6 levels were detected in platinum-based resistant and sensitive cases, they reported lower ascitic IL-6 in patients who responded to chemotherapy, although the difference was not found to be statistically significant.

In EOC, ascites is present in at least one third of patients and is believed to contribute to the spread of cancer to secondary sites [32], as well as to peritoneal dissemination [33]. Interestingly, the predictive value of IL-6 for the development of post-treatment ascites was observed in the present study. In line with our findings, Plante el al [20] reported a direct association between ascitic IL-6 and ascites volume. In agreement, Lo et al. [8] described an IL-6 trans-signaling pathway in formation and progression of ascites in EOC wherein IL-6 and its soluble IL-6 receptor (IL-6Rα) serve as an important regulator of endothelial cell survival, migration, and integrity, and IL-6 trans-signaling on endothelial cells prevents chemotherapy-induced apoptosis, induces endothelial hyperpermeability, and increases transendothelial migration of ovarian cancer cells, contributing to ascites formation and tumor progression.

Future clinical investigations are needed to establish improved clinical evidence to guide patient care in EOC. With respect to the results from the present study, these investigations could be developed at two levels: 1) the assessment of the effectiveness of IL-6 as a tool for post-treatment monitoring of EOC patients, alone or in combination with current biomarkers in use; 2) the evaluation of IL-6 as a potential target in the treatment of EOC. With regard to its value as a potential target for developing novel treatment strategies, our laboratory has previously studied the inhibitory effects of minocycline on IL-6 production in EOC. It was shown in in vivo models of EOC that orally administered minocycline was highly effective in decreasing tumor burden and suppressing ovarian cancer-induced malignant ascites by targeting IL-6 [34].

## Conclusions

Here, we report the upregulation of IL-6 in EOC, with predictive value for development of ascites following the platinum-based treatment. In keeping with results from previous studies, our findings highlight the role of this cytokine in EOC patients which needs to be further explored in future research.

## Abbreviations

DFS: Disease-free survival
EOC: Epithelial ovarian cancer
IL-6: Interleukin-6
OS: Overall survival
